# Attitudes about Future Genetic Testing for Posttraumatic Stress Disorder and Addiction among Community-Based Veterans

**DOI:** 10.3389/fpsyt.2017.00076

**Published:** 2017-05-15

**Authors:** Michelle R. Lent, Stuart N. Hoffman, H. Lester Kirchner, Thomas G. Urosevich, Joseph J. Boscarino, Joseph A. Boscarino

**Affiliations:** ^1^Department of Epidemiology and Health Services Research, Geisinger Clinic, Danville, PA, USA; ^2^Geisinger Clinic, Danville, PA, USA; ^3^Department of Biomedical and Translational Informatics, Geisinger Clinic, Danville, PA, USA; ^4^William James College, Newton, MA, USA

**Keywords:** genetic testing, posttraumatic stress disorder, addiction, veterans, psychiatric genetics

## Abstract

This study explored attitudes toward hypothetical genetic testing for posttraumatic stress disorder (PTSD) and addiction among veterans. We surveyed a random sample of community-based veterans (*n* = 700) by telephone. One year later, we asked the veterans to provide a DNA sample for analysis and 41.9% of them returned the DNA samples. Overall, most veterans were not interested in genetic testing neither for PTSD (61.7%) nor for addiction (68.7%). However, bivariate analyses suggested there was an association between having the condition of interest and the likelihood of genetic testing on a 5-point scale (*p* < 0.001 for PTSD; *p* = 0.001 for alcohol dependence). While ordinal regressions confirmed these associations, the models with the best statistical fit were bivariate models of whether the veteran would likely test or not. Using logistic regressions, significant predictors for PTSD testing were receiving recent mental health treatment, history of a concussion, younger age, having PTSD, having alcohol dependence, currently taking opioids for pain, and returning the DNA sample during the follow-up. For addiction testing, significant predictors were history of concussion, younger age, psychotropic medication use, having alcohol dependence, and currently taking opioids for pain. Altogether, 25.9% of veterans reported that they would have liked to have known their genetic results before deployment, 15.6% reported after deployment, and 58.6% reported they did not want to know neither before nor after deployment. As advancements in genetic testing continue to evolve, our study suggests that consumer attitudes toward genetic testing for mental disorders are complex and better understanding of these attitudes and beliefs will be crucial to successfully promote utilization.

## Introduction

Understanding of the genetic bases of psychiatric disorders continues to improve, despite the complexity and various gene–environment interactions that can influence such behaviors (1). The potential benefits of genetic testing may include better diagnostic screening, early prevention and, in the case of Mendelian genetic disorders, the ability to make more informed reproductive decisions (2, 3). Conversely, genetic testing could impact well-being (4, 5), raise the potential for discrimination (2, 3), and cause conflict within families (4). Questions surrounding privacy, as well as who should be tested and why, also present multifaceted dilemmas (2, 4). Genetic testing for psychiatric disorder risk is likely to become increasingly incorporated into clinical practice in the future, especially as more direct-to-consumer testing products become available (6).

A review of studies on general consumer attitudes surrounding genetic testing for psychiatric disorders identified several areas for future study: specifically, the need to study individuals without psychiatric disorders or not involved in genetics-related research, and the need for larger samples (2). Only one study to date has evaluated veterans’ attitudes toward psychiatric disorder genetic testing (7). This study found that veterans with posttraumatic stress disorder (PTSD) viewed genetic testing for PTSD less favorably than those without PTSD and were more likely to fear insurance discrimination.

Attitudes toward genetic testing for psychiatric disorders among military veterans are largely unknown despite the high prevalence of certain psychiatric disorders among veterans and the regular screening of patients at VA facilities for psychiatric disorders, including PTSD and addiction (8, 9). Prevalence estimates of PTSD range from 10% in male Gulf War veterans to 15% in Vietnam-era male veterans, compared to 3.6% of males in the general population (10–13). Additionally, 2 out of every 10 veterans with PTSD also live with substance use disorders (13).

Consumer attitudes toward genetic testing for psychiatric disorders are mixed (3, 5, 7). In one study, 83% of family members of individuals with schizophrenia reported interest in genetic testing for this condition (14). Additionally, individuals with a family history of bipolar disorder generally endorsed that they would pursue genetic testing if the assessment has high diagnostic certainty, but fewer wanted their children tested at birth (3). Unaffected relatives of individuals with psychiatric disorders emphasized the importance of genetic counseling prior to testing to provide consumers with an accurate assessment of potential risk in the context of reproductive decision-making (15). Consumers with psychiatric disorders not only reported interest in contributing to genetic research but also reported concerns regarding having to make “difficult choices,” the perception of certain lives as more valuable than others, and the possibility of discrimination (5). Individuals with alcohol use disorders were willing to provide genetic samples but also expressed concerns surrounding health insurance denial, employment discrimination, and identity theft (16).

In this study, we randomly surveyed a cohort of 700 community-based U.S. military veterans with and without psychiatric disorders regarding their attitudes about hypothetical genetic testing at baseline for PTSD and addiction. All study veterans served at least one warzone deployment and were considered to be at risk for these conditions post deployment (17). One year later, we recontacted these veterans and asked them to return a DNA sample for genetic analyses.

## Materials and Methods

### Participants

The study cohort included a random sample of community-based U.S. military veterans (*n* = 700) recruited as part of a broader study of the health effects of military service (18). For our sample, we created a computer-generated random number, which was then assigned to each veteran identified in our Health System. Based on this number, patients were randomly selected for survey interviews. All participants were outpatients in the multihospital system and self-identified as veterans, which was verified during the survey recruitment. Veterans with and without psychiatric disorders were interviewed.

Inclusion criteria were English-speaking, had one or more deployments in a warzone, and had an age less than 75 years. Institutionalization and an inability to complete a 45-min interview were exclusion criteria. The study participation rate was estimated to be 65% (19). The Health System’s Institutional Review Board approved the study protocol, and all participants provided verbal informed consent for the baseline phone survey and written consent for the DNA sample at follow-up. A small incentive was provided to complete the survey ($15) and to return the DNA sample by mail ($15).

### Telephone Interviews

The interviews were administered by telephone by trained mental health interviewers working in a supervised, professional survey research center from December 2011 through January 2012. The survey instruments utilized were used in previous studies (20–25) and validated in national and regional trauma studies involving over 30,000 respondents exposed to psychological trauma (24, 26). Comparison of telephone vs. in-person surveys using survey instruments suggests similar results in mental health studies (27, 28).

Participants provided information on military service branch, deployment history, smoking history, mental health history, concussion history, and other demographic and medical information (18). They were also screened for probable alcohol dependence using the CAGE (Cut Down, Annoyed, Guilty, and Eye Opener) questionnaire (29). Combat exposure was assessed using a version of the Combat Experience Scale (30).

Participants answered questions regarding their attitudes toward hypothetical genetic testing at baseline for PTSD and addiction after the following introduction was provided: “Genetic tests are being developed for different medical problems. Soon genetic tests may be available to predict a person’s risks for addiction and posttraumatic stress disorder. If such genetic tests were available, how likely would you be to have this genetic test?” This question was assessed on a 5-point Likert Scale, ranging from “extremely likely” to “not likely at all.” Following this question, they were asked two open-ended questions about their answers to these Likert-scaled questions (i.e., “Why did you respond like you did for addiction?”). Participants were also asked when they would have liked to have known their genetic risks (i.e., before deployment, after or neither before or after deployment). Measures of trust, discrimination, and concerns about privacy were not explicitly assessed in the current survey. However, we did use open-ended survey questions as a way to capture the concerns of veterans, as is commonly done in behavioral research (31, 32). We inquired about health insurance status, employment status, and use of VA services to add to our analyses to assess the impact of these variables on testing responses.

Our open-ended questions were administered and coded in accordance with standard procedures for survey research (31, 32). Open-ended questions were recorded verbatim during the interview. Each question was probed up to three times for specificity, if necessary. For instance, if a vague response was given (e.g., “it depends …”), the interviewer probed the respondent to be more specific. After survey completion, the verbatim responses were manually coded. Following this, tabulations were compiled and coding was revised in consultation the with study Principal Investigator to capture any new categories that emerged or to collapse existing categories (31, 32).

### DNA Sample Collection

Approximately 1 year after the survey, veterans were recontacted and asked to provide a DNA sample using Orogene DNA kits manufactured by Genotek, Inc. (Ottawa, ON, Canada). Those who agreed to test where mailed a saliva kit with instructions to complete and return to the study team using a postage-paid return envelope. Altogether, 41.9% (95% CI = 38.2–45.6) of veterans returned their DNA kits to the study team.

### Statistical Analyses

Descriptive statistics characterized the study sample. Likelihood of testing was analyzed both as an ordinal scale and collapsed into binary outcome variables (extremely likely, very likely, and likely = yes; not very likely, not likely at all = no) for PTSD and addiction, respectively, based on our statistical results. Since the dichotomous measures demonstrated the best statistical fit (33, 34), we present these results in the current paper. Next, multivariable logistic regressions (33), using stepwise backwards elimination were used with the *p*-value ≥0.05 for variable elimination in order to identify the best predictor variables associated with the likelihood of proving a DNA sample for testing. Our candidate predictor variables included multiple tours, poor/fair current health, high combat exposure, age, education, race, sex, being employed, having health insurance, having current PTSD, having current depression, using the VA for health care, having a positive current screen for alcohol dependence, using mental health services in past year, a history of concussion, using psychotropic medications in past year, using painkillers in past year, and returning the DNA sample at follow-up. Verbatim responses were manually coded by study staff and grouped categorically. “Don’t know” responses for the testing questions (<1%) were deleted for the purposes of regression analyses. Statistical analyses were conducted using Stata, version 13.1 (Stata Corp, College Station, TX, USA, 2014).

## Results

### Sample

Most veterans were between 40 and 64 years old (59.3%), male sex (95.9%), white race (93.3%), married (79.6%), had some college or higher education (57%), had health insurance (96.3%), were Vietnam veterans (72%), and a significant number (40.4%) currently used the VA health-care system (Table 1). The majority had a history of cigarette smoking (72.1%), and approximately 12% had a recent history of alcohol misuse (heavy drinking, 11.9%; positive alcohol dependence results on the CAGE scale, 12.4%).

**Table 1:** **Sample characteristics (*N* = 700)**.

Study variables	*n* (%)
**Age**	
18–39	66 (9.4)
40–64	415 (59.3)
65+	219 (31.3)
Male	671 (95.9)
White race	653 (93.3)
Married	557 (79.6)
Employed full-time or part-time	313 (44.7)
Have health-care insurance	674 (96.3)
Some college or higher	399 (57.0)
Multiple warzone deployments	200 (28.6)
Currently use VA for health care	283 (40.4)
Received mental health treatment, past year	153 (21.9)
Reports poor/fair current health	221 (31.6)
Returned DNA sample	293 (41.9)
**Warzone service**	
Vietnam	504 (72.0)
Gulf war	68 (9.7)
Iraq/Afghanistan	96 (13.7)
Other	32 (4.6)
**Substance use/medication use/combat**	
Smoked 100+ cigarettes in lifetime	505 (72.1)
Drank nearly every day in past year	66 (9.4)
Heavy drinking in the past 30 days	83 (11.9)
Alcohol dependence in past year^a^	87 (12.4)
Used psychotropic medications in past year	144 (20.6)
Used prescription painkillers in past year	97 (13.9)
Combat was major traumatic event	569 (82.3)
High combat exposure history	148 (21.1)
History of concussion during deployment	181 (25.9)
**Psychiatric disorders**	
Lifetime posttraumatic stress disorder (PTSD)	67 (9.6)
PTSD in past year	47 (6.7)
Subclinical PTSD in the past year^b^	109 (15.6)
Lifetime major depression disorder	129 (18.4)
Major depression in past year	44 (6.2)

*^a^CAGE questionnaire (29)*.

*^b^Breslau criteria for PTSD (35)*.

### Mental Health History

A significant number of veterans were classified as having high combat exposure (21.1%), lifetime PTSD (9.6%), lifetime major depression (18.4%), poor or fair current health (31.6%), and having used prescription opioids in the past 12 months (13.9%). Approximately 20% of veterans were taking psychotropic medications (20.6%), had a recent mental health-related visit (21.9%), or had a history of concussion during their deployment (25.9%). In addition, more than 80% of veterans (82.3%) reported that being in combat was a major traumatic event for them (Table 1).

### Attitudes about Genetic Testing

The majority of participants responded that they were “Not Likely at All,” or “Not Very Likely” to pursue genetic testing for PTSD (61.7%, Table 2) or for addiction (68.7%). Additionally, 25.9% would have wanted to know about their genetic risk before deployment, 15.6% after deployment and 58.6% did not want to know their genetic risk before or after deployment (Table 2). While bivariate associations were found for current PTSD and addiction and the likelihood of testing based on Spearman corrections (*p* < 0.001 for PTSD; *p* = 0.001 for addiction), the ordinal logistic regression results for these analyses fit poorly (Pseudo *R*^2^ = 2% for addiction; Pseudo *R*^2^ = 6% for PTSD). Consequently, we collapsed these results into binary “yes/no” measures and used logistic regressions for these analyses.

**Table 2:** **Veterans’ interest in genetic testing for posttraumatic stress disorder (PTSD) and addiction (*N* = 700)**.

Testing interest/attitudes	*n* (%)	Yes/no, *n* (%)^a^
**Genetic testing for PTSD**		
Extremely likely	75 (10.7)	Yes, 270 (38.6)
Very likely	77 (11.0)	
Likely	118 (16.9)	
Not very likely	93 (13.3)	No, 427 (61.7)
Not likely at all	334 (47.4)	
Don’t know/uncertain	3 (0.4)	
**Genetic testing for addiction**		
Extremely likely	60 (8.6)	Yes, 213 (30.4)
Very likely	54 (7.7)	
Likely	99 (14.1)	
Not very likely	87 (12.4)	No, 481 (68.7)
Not likely at all	394 (56.3)	
Don’t know/uncertain	6 (0.9)	
**Would liked to have known genetic results**		
Before deployment	181 (25.9)	
After deployment	109 (15.6)	
Neither before or after deployment	410 (58.6)	

*^a^Yes = extremely likely, very likely, and likely. No = not very likely, not likely at all*.

In veterans who did endorse interest in genetic testing for PTSD or addiction, the final stepwise logistic regression models found seven significant predictors for the likelihood of PTSD testing and five predictors for addiction testing (Table 3). For PTSD testing, receiving recent mental health treatment (OR = 2.30, CI = 1.51–3.52, *p* < 0.001), history of concussion (OR = 2.01, CI = 1.37–2.93, *p* < 0.001), younger age (OR = 0.97, OR = 0.96–0.99, *p* < 0.001), current PTSD (OR = 4.07, CI = 1.80–9.20, *p* = 0.001), current alcohol dependence (OR = 2.12, CI = 1.29–3.50, *p* = 0.003), use of opioids for pain (OR = 1.69, CI = 1.04–2.74, *p* = 0.033), and returning the DNA sample at follow-up (OR = 1.41, CI = 1.01–1.99, *p* = 0.047) were associated with the likelihood of genetic testing for PTSD at baseline. For addiction testing, history of concussion (OR = 1.71, CI = 1.18–2.48, *p* = 0.004), younger age (OR = 0.98, CI = 0.97–0.99, *p* = 0.005), currently taking psychotropic medications (OR = 1.57, CI = 1.04–2.35, *p* = 0.030), current alcohol dependence (OR = 1.85, CI = 1.15–2.98, *p* = 0.011), and the use of prescription opioid painkillers in the past year (OR = 1.73, CI = 1.09–2.76, *p* = 0.021) were associated with the likelihood of genetic testing for addiction.

**Table 3:** **Stepwise logistic regressions predicting genetic testing (*N* = 700)**.^a^

Predictor variables	OR (95% CI)	*p*-Value
**Yes, interest in genetic testing for posttraumatic stress disorder (PTSD)^b^^,^^d^**		
Age (years)	0.97 (0.96–0.99)	<0.001
Current alcohol dependence^c^	2.12 (1.29–3.50)	0.003
Current PTSD	4.07 (1.80–9.20)	0.001
Mental health treatment past year	2.30 (1.51–3.52)	<0.001
History of concussion during deployment	2.01 (1.37–2.93)	<0.001
Used prescription painkillers in past year	1.69 (1.04–2.74)	0.033
Returned DNA sample	1.41 (1.01–1.99)	0.047
**Yes, interest in genetic testing for addiction^b^^,^^e^**		
Age (years)	0.98 (0.97–0.99)	0.005
Current alcohol dependence^c^	1.85 (1.15–2.98)	0.011
Used psychotropic medications in past year	1.57 (1.04–2.35)	0.030
Used prescription painkillers in past year	1.73 (1.09–2.76)	0.021
History of concussion during deployment	1.71 (1.18–2.48)	0.004

*^a^Variables assessed in backward stepwise logistic regression included serving multiple tours, having poor/fair health, high combat exposure, age, having some college or more, white race, male sex, employed full-time/part-time, having health insurance, current PTSD, current depression, currently using VA, having a positive screen for alcohol dependence, used mental health services in past year, having history of concussion, used psychotropic medications in past year, used painkillers in past year, and return of the DNA sample at follow-up. Variables with p ≥ 0.05 were removed from the analysis*.

*^b^Yes = extremely likely, very likely, likely. No = not very likely, not likely at all*.

*^c^CAGE questionnaire (29)*.

*^d^Area under ROC = 0.71; Hosmer–Lemeshow χ^2^ = 5.72, df = 8, p = 0.68; Pseudo R^2^ = 0.12*.

*^e^Area under ROC = 0.65; Hosmer–Lemeshow χ^2^ = 8.79, df = 8, p = 0.36; Pseudo R^2^ = 0.05*.

The results for the open-ended reasons given for both interest in and no interest in genetic testing for PTSD and addiction, respectively, are shown in Table 4. Reasons given for interest in genetic testing for both PTSD and addiction were mostly related to self-benefit and gaining additional knowledge (for PTSD, 25 + 22 = 47%; for addiction, 29 + 24 = 53%). Reasons given for *not* testing are related to not having the condition or being reported as unnecessary (for PTSD, 31 + 19 = 50%; for addiction 42 + 14 = 56%). Concerns about privacy, confidentiality, discrimination, insurance coverage, etc., were not mentioned by the veterans.

**Table 4:** **Open-ended reasons given for interest/no interest in genetic testing for posttraumatic stress disorder (PTSD)/addiction**.

**Reasons given for PTSD testing (*n* = 267)**
Curious/would like to know (25%) Self-benefit/knowledge (22%) Already diagnosed (15%) To help others (11%)
**Reasons given for no PTSD testing (*n* = 417)**
Does not have PTSD (31%) Unnecessary (19%) Older age (18%)
**Reasons given for addiction testing (*n* = 210)**
Self-benefit/knowledge (29%) Curious/would like to know (24%) Family history (13%)
**Reasons given for no addiction testing (*n* = 470)**
No addiction history (42%) Unnecessary (14%) Older age (13%)

## Discussion

The principal study finding was that the majority of veterans were not interested in hypothetical genetic risk testing for PTSD (62%) or addiction (69%) at baseline. Most veterans (58.6%) reported that they would not want to know their genetic status either before or after deployment. The most frequent reasons for lack of interest were not feeling at risk for these disorders and questioning the utility of genetic testing. Participants did not mention trust, ethical issues, or concern about employment. However, the majority of the veterans surveyed (55.3%) were not employed and were over 40 years old (90.6%). In addition, 96.3% reported currently having insurance coverage. Previously, challenges trusting researchers, privacy, discrimination concerns, and questions as to how genetic information would assist in psychiatric treatment were cited as reasons for testing hesitancy (5, 7).

Having current PTSD increased the likelihood of interest in genetic testing for PTSD fourfold (OR = 4.07). Recent mental health treatment, younger age, a history of concussion, a positive screen for alcohol dependence, a use of opioid pain medicines, and having returned the DNA sample at the follow-up, also was associated with interest in genetic testing for PTSD. Our findings differ from a previous study that found veterans in the VA system with PTSD expressed less favorable attitudes toward genetic testing than those without PTSD (7). Our larger sample size, older cohort, and our non-VA-based sample may account for the differences in results. For addiction, history of concussion during deployment, younger age, taking psychotropic medications, a positive alcohol dependence screen, and recent use of opioids for pain were associated with increased interest in testing.

Genetic testing for psychiatric disorder risk would be most useful if testing occurred before the onset of symptoms, or specifically in veterans, before combat exposure. Testing has the potential to promote the practice of precision medicine (36) by informing prevention approaches at the individual level. Early testing could identify and target individuals at high risk via programs that include psychoeducation and heightened surveillance (37). However, participants in our study expressed little interest in genetic testing for two psychiatric disorders, citing often that they did not have that problem or condition. Future studies could evaluate whether interest in genetic testing for PTSD or addiction increases when tests are available to the patient or occurs at a younger age. When participants did endorse interest in genetic testing, reasons cited were most commonly for self-benefit and gains in knowledge. Highlighting these potential benefits of testing could improve interest and participation. Also noteworthy is that the current study was a hypothetical assessment, since no genetic testing was actually available at the time of the baseline survey.

Genetic counseling, including education surrounding the clinical utility of genetic testing and the protections afforded by the Genetic Information Non-discrimination Act (38), may increase interest in testing; however, privacy and discrimination were not issues for veterans in the current study. Moreover, counseling may move patients closer toward acceptance of these disorders, leading to greater treatment-seeking behaviors (39). Counseling prior to testing can help to mediate misconceptions and fears surrounding uncertainty (40), perceived risk (41, 42), and stigma (43) and improve knowledge about causes of psychiatric disorders (41, 43). However, for conditions such as PTSD and addiction, which are complex and multifactorial disorders, genetic counseling is limited at this time because the genetic variants known to date only explain a small percentage of the phenotypic variance (37, 44). Nevertheless, the epidemiology, biology, diagnosis, associated comorbidity, and PTSD treatment options are considered well understood at this time (24, 26, 45, 46).

To date, our study is the largest investigation of attitudes toward psychiatric genetic testing and included individuals with and without psychiatric disorders who were not already engaged in genetic testing. The prevalence of psychiatric disorders in our community-based veteran sample resembled the general U.S. population more than many VA-based cohorts (18), potentially expanding the applicability of our findings. One limitation of this study was that our cohort was largely homogenous (>90% male, White, and older) Vietnam-era veterans (72%), which may not represent younger or female veterans’ views toward genetic testing for PTSD and addiction. Future studies should include more diversity and evaluate a wider range of psychiatric disorders.

In summary, the majority of veterans did not express interest in genetic testing for PTSD or addiction, two psychiatric disorders of particular relevance to this population and that are of high interest in the VA system and to the veteran community (18, 46). As prospective discoveries and subsequent advancements continue to improve genetic testing, a more comprehensive understanding of views regarding screening will become increasingly imperative to expand its clinical application. It is important to emphasize that PTSD, in particular, is not just another mental disorder for veterans but also a compensated medical condition associated with military service and war-fighting worldwide.

## Ethics Statement

The health system’s Institutional Review Board approved the study protocol, and all participants provided informed consent.

## Author Contributions

JAB, JJB, SH, HK, and TU are responsible for study conception, design, and implementation. JJB assisted with analysis and manuscript writing. HK, ML, and JAB conducted data analysis. ML wrote the first draft of the manuscript. All the authors reviewed and approved the final manuscript.

## Conflict of Interest Statement

The authors declare that the research was conducted in the absence of any commercial or financial relationships that could be construed as a potential conflict of interest.
